# Usefulness of Maternal and Fetal Parameters for the Prediction of Parturition Date in Dogs

**DOI:** 10.3390/ani11030878

**Published:** 2021-03-19

**Authors:** Giulia Siena, Chiara Milani

**Affiliations:** Department of Animal Medicine, Production and Health (MAPS), University of Padua, 35020 Legnaro, PD, Italy; giulia.siena@phd.unipd.it

**Keywords:** dog, pregnancy, parturition, maternal parameters, fetal parameters, ultrasound, fetal maturity

## Abstract

**Simple Summary:**

Nowadays, the scientific literature lists several parameters and formulas for the prediction of parturition date in dogs, and their accuracy is affected by various factors: pregnancy phase, litter and also maternal size. The selection of the most appropriate parameter set for ultrasound assessment of gestational age and fetal organ development is essential to predict parturition date with high accuracy, but the high number of factors influencing these parameters challenges their clinical interpretation. In this review, the variables of interest are grouped as (i) maternal parameters, (ii) fetal parameters, (iii) ultrasonographic assessment of maternal and fetal heart rate and blood flow, and (iv) markers of fetal maturity. The aim of this review is to analyze the parameters that predict parturition date in the canine species, especially their accuracy, and finally propose to consider the evaluation of fetal maturity as mark of the readiness for parturition combined with the other most studied parameters.

**Abstract:**

An accurate parturition timing is of key importance for breeders and veterinarians in order to give professional assistance to parturition in dogs. However, pregnancy length calculated from the breeding date has a wide variability. Different parameters and formulas have been described and calculated, as well as their accuracy which is affected by various factors: stage of pregnancy, litter and maternal size. Therefore, the selection of the most appropriate parameter panel poses the challenge of weighing their influences and impact on the overall accuracy. The aim of this review is to analyze the parameters useful for parturition timing, especially their accuracy, and to propose the addition of fetal maturity and criteria for its evaluation to detect readiness for parturition. Parameters, as described in literature, are classified as: (i) maternal parameters, (ii) fetal parameters, (iii) ultrasonographic assessment of maternal and fetal heart rate and blood flow, (iv) parameters indicating fetal maturity. A focus on recently described parameters—such as fetal gastrointestinal motility and fetal lung development detected by quantitative ultrasound—is reported. Currently, the most accurate way to predict parturition day is represented by a prepartum progesterone drop, but the identification of a panel of ultrasonographic parameters combining their significance and their accuracy throughout pregnancy is still needed.

## 1. Introduction

In small animals, neonatal mortality is an event which represents a potential emotional and economic loss for pet owners and breeders. An accurate prediction of the parturition day could be useful for both owners and veterinarians, to allow better planning for labor assistance and potential cesarean section (C-section) so as to improve neonatal survival and minimize neonatal death. In dogs, the breeding date is not accurate for predicting parturition because a physiological pregnancy may last from 57 to 72 days from the breeding date [1,2,3,4]. In the last decades, many studies have proposed different methods for the estimation of parturition date [1,5,6,7,8,9,10,11,12,13]. More recently, parameters and formulas have been described and calculated using ultrasound (US), showing a different accuracy mainly due to pregnancy phase and litter size [14,15,16,17,18,19].

The aim of this review is to analyze the parameters described in the literature for the prediction of parturition date in dogs. These parameters are classified as maternal parameters, fetal parameters, ultrasonographic assessment of maternal and fetal heart rate and blood flow, with a focus on their accuracy and on the factors influencing them, and to propose the evaluation of fetal maturity as a sign of readiness for parturition combined with other most studied parameters.

## 2. Factors Influencing Pregnancy Length

Pregnancy length from ovulation to parturition seems not to be affected by sex ratio of puppies, age of the bitch, regularity of interestrous intervals, parity, type of mating (assisted or natural) and type of semen used [20]. Litter size, breed and maternal size seems to affect gestational length [20,21], although this was not evidenced by all authors [6,12,22,23].

Singleton or two-puppy pregnancies are reported to be longer than those with more than three puppies (65.2 ± 2.6 days for ≤2 puppies, 62.8 ± 1.9 for 3–9 puppies, 62.2 ± 1.3 days for >10 puppies, respectively) [20]. In large-size bitches, pregnancies are reported to be on average one day longer when carrying four or fewer puppies, compared to those with five or more puppies (65.2 ± 2.9 days and 63.5 ± 1.9 days, respectively) [21]. In a retrospective study on Drever bitches, an increase or a decrease of 0.25 days in length was observed in pregnancies with a litter size lower or greater than the mean number of pups (6–8 puppies/litter), respectively [24].

The results available in the literature about the relationship between pregnancy length and maternal size and breed are controversial [20,21,25,26]. In a retrospective study taking into account 162 pregnancies, small-size bitches (<10 kg) had a gestational length shorter than large and giant size bitches (25–40 kg and >40 kg, respectively) [20]. However, in West Highland White Terriers (WHWT), the gestational period was reported to be longer than in German Shepherd, Labrador Retriever and Doberman bitches (62.8 ± 1.2, 60.4 ± 1.7, 60.9 ± 1.5, 61.4 ± 1 days, respectively) [25,27]. On the other hand, Cavalier King Charles Spaniels (CKCS), which have a similar size to WHWT, had a gestational period shorter than that of German Shepherds, Golden Retrievers and Rottweilers (61 ± 1.5, 63.2 ± 1.8, 64.3 ± 1.3, 65.6 ± 1.6 days from ovulation to parturition, respectively), as well as the other 18 different breeds considered (62.8 ± 2, 64.5 ± 1.4 days, for CKCS vs. other breeds from LH peak to parturition, respectively) [20,26]. Pregnancy of German Shepherd bitches is shorter than Boxers, Bernese Mountain dogs, Old English Sheep Dogs and Bouvier des Flandres (60.4 ± 1.7, 62.4 ± 0.7, 61.7 ± 0.3, 62 ± 0.3, 62.3 ± 0.4 days from mating established measuring P4 to parturition, respectively) [25,27]. Eilts et al. [21] found that in German Shepherd, Golden Retriever and Hounds bitches, the gestational period is longer than in Labrador Retrievers (63.6 ± 2.1, 64.7 ± 1.5, 66 ± 2.8, 62.9 ± 1.3 days from LH peak or for Hounds form D1 + 8 days to parturition, respectively) [21].

The correlation between pre-gestational body weight and gestational length requires further investigation. Some authors have suggested a correlation between them [1]; however, a broader analysis taking into account both breeds and pre-gestational body weight is still lacking. The influence of breed can be masked by the maternal size effect, because many studies make a comparison considering weight classes and not breeds, and because a 0.8 days of difference may be due to the assay technique and reference range used to estimate the day of ovulation [20].

## 3. Maternal Parameters Used for the Prediction of Parturition

### 3.1. Vaginal Cytology

In the bitch, a standard protocol for the collection of samples, staining method and evaluation criteria of vaginal cytology has not been fully adopted; therefore, considerable variability in its interpretation exists between veterinarians and authors when staging the estrous cycle [28]. Vaginal cytology should not be used for ovulation timing on its own, and it is not indicative of the serum progesterone (P4) concentration [1,28]. The use of vaginal cytology for a continuous monitoring of the reproductive cycle could instead be helpful in determining the proper time to start serum P4 monitoring [28].

However, vaginal cytology is useful for detecting the first day of diestrus (D1), after which pregnancy will last 57 ± 3 days (54–60 days) [3,29]. The easiness of the identification of D1 is due to a change in the percentage of cornified cells which from the 80–100% decrease of at least 20%, and the appearance of parabasal cells and neutrophils in less than 24–48 h. D1 is defined as the first day in which this abrupt drop is evident. A recent study investigated the accuracy of the identification of cytological diestrus for parturition timing [29]. In this study, the D1 identification resulted in being a reliable parameter for the determination of parturition onset within 57 ± 1, ±2 and ±3 days in 88%, 99% and 100% in the 242 monitored pregnancies. In 95% of cases, the interval between D1 and the day of cervical dilatation varied between 55 and 59 days. This interval could be affected by breed and litter size, but these variations were not of clinical significance. Therefore, the identification of D1 with the help of vaginal cytology could be an interesting method for parturition timing because it is cheap, rapid and reliable [29].

### 3.2. Progesterone Assay

#### 3.2.1. Progesterone Assay for the Identification of the Ovulation Day

Serum P4 concentration is an essential parameter for the detection of the LH peak, ovulation and parturition date. Serum P4 concentration begins to rise before the onset of the luteal phase, and the first day in which P4 is ≥1.5 ng/mL is considered to indicate the LH peak [1]. Other authors considered indicative of the LH peak a P4 concentration of 2–3 ng/mL [20] or a P4 ≥ 1.5 ng/mL when followed by a P4 ≥ 3 ng/mL on the following day [2].

On the ovulation day, which occurs almost 48–60 h after the LH peak, P4 concentration reaches values between 4–10 ng/mL [4,11]. In other studies, P4 concentration at ovulation is reported to be between 3.4 and 6.6 ng/mL or when it reaches a value of 6 ng/mL [20,22]. From ovulation, pregnancy length is reported to be 63 ± 1 days (62–64 days) [4,30]. Kutzler et al. [1] found that the slope of the initial rise of P4 concentration from the first day after LH peak was significatively influenced by pre-gestational body weight, with a sudden increase in small size dogs compared to medium and in large size dogs compared to giant. The accuracy of parturition timing using pre-breeding P4 concentration is described to be 67, 90 and 100% within 65 ± 1, ±2 and ±3 days, respectively, and this accuracy is not affected by litter size or maternal body weight [1].

#### 3.2.2. Progesterone Assay in the Prepartum Period

In the prepartum, an abrupt decrease in serum P4 concentration occurs, reaching a value lower than 2 ng/mL (about 6.4 nmol/L) in the last 24 h before parturition [1,3,4]. Another study reported that a serum P4 concentration drop from 4–10 ng/mL to values < 2.0 ng/mL in 12–24 h indicates parturition within 1 or 2 days [31]. Serum P4 concentration is less than 1 ng/mL at parturition [2,3,5,32,33,34]. This rapid decline is due to the PGF2alfa release and the consequent luteolysis that begins 36 h before parturition [31]. A negative correlation between prostaglandin F2alpha (PGF2alpha) and P4 concentration was found in the prepartum in the bitch [5]. In singleton pregnancy, it could be possible that P4 concentration will not drop below 2.0 ng/mL at term, probably due to incomplete luteolysis [3,34].

Using the radioimmunoassay (RIA) method, serum P4 concentration observed during the last 5 days of pregnancy declines from a mean value of 4.5 ± 0.6 ng/mL (range from 2.6–7.8 ng/mL) at day −5 to 3.12 ± 0.40 ng/mL 40–32 h before parturition [35]. In the last 24 h prepartum, P4 is 1.19 ± 0.36 ng/mL, with a further decrease to 0.55 ± 0.07 ng/mL during the last 12–8 h prior to parturition. On the day of parturition, P4 concentration was basal and remained below 0.5 ng/mL [35]. Another study reported P4 values of 9.3 ± 5.6 nmol/L (2.92 ± 1.76 ng/mL) at 24 h prepartum and a lower value of 1.6 ± 0.7 nmol/L (0.50 ± 0.22 ng/mL) at 24 h postpartum [5].

The chemiluminescent immunoassay (CLIA) method was used to assess the diagnostic efficacy of a single P4 measurement in the prepartum in the bitch [9]. Rota et al. [9] reported that a P4 concentration below 3.4 ng/mL indicates bitches who will deliver the following day, with a low sensitivity (46.88%) and a high specificity (100%). Rota et al. [9] concluded that, because of individual differences, a single P4 assay in the prepartum has a low diagnostic efficacy, whereas it could be used as a first selection method to discriminate the bitches who are at the end of pregnancy and on which a C-section could be performed from those who are not at term. In a recent study, other cut-off values for a single prepartum P4 assay with a RIA method have been reported [36]. De Cramer and Nöthling [36] found that bitches with a P4 value < 8.7 nmol/L (2.74 ng/mL) had a 99% of probability to present cervical dilatation during the following 48 h and bitches with a P4 value < 3.4 nmol/L (1.07 ng/mL) had a 100% probability of presenting cervical dilation, indicating readiness for parturition during the following 24 h. The authors suggest that the 94% of bitches with a serum P4 value higher than 8.7 nmol/L (2.74 ng/mL) are not expected to be ready to deliver during the following 12 h [36].

### 3.3. Rectal Temperature

Body temperature is related to circadian rhythm and serum P4 concentration during the prepartum period in the bitch [36,37]. Progesterone is likely to be a thermogenic hormone, and its sudden decrease due to prepartum luteolysis could determine a rectal temperature drop greater than the circadian rhythm variations [7,36,37]. To detect a significant prepartum variation in rectal temperature, a temperature baseline should be defined, and for this reason it should be measured at least twice a day during the last 14 days of pregnancy or from day 55 after mating [3,38,39]. Temperature declines by nearly 1 °C in the last 12–24 h of pregnancy, following the abrupt decrease of P4 [9,31,38]. In the last 6-18 h prior to parturition, temperature could decrease from 1.1 to 1.7 °C below the baseline [3]. Some difficulties in the assessment of body temperature drop have been reported, due to different confounding factors like inter- or intra-observer variability, as well as thermometer and penetration depth variability [40]. In small-size breeds, body temperature could reach lower values (as low as 35 °C) than in large-size breeds (as low as 37 °C) [3].

To the best of authors’ knowledge, no papers reporting the predictive value of rectal temperature measurement in relation to parturition timing have previously been published, with the exception of one proceeding abstract that reported a decrease of 0.4 °C indicating parturition within 24 h with predictive negative and positive values of 99.3% and 25.2%, respectively [39]. In conclusion, the reliability of rectal temperature as a predictor for the parturition date is controversial. Rectal temperature drop could not be detected in all bitches and its threshold value could be very variable; therefore, it should not be used as a unique parameter for parturition or C-section timing [9,36,38,41].

### 3.4. Vaginal Temperature

Vaginal temperature may be considered, combined with other parameters, for parturition timing during the last week of pregnancy, because of its drop in the last 48 h prior to parturition [41]. Vaginal temperature has been described to be significantly correlated with rectal temperature in the canine species [41,42]. It can be assessed using a thermometer or a validated vaginal temperature logger measuring body temperature every 10 min, placed 14–18 cm deep into the vagina of the bitch during the last 3–8 days of pregnancy, or from day 56–61 after the first mating [7,42]. To have results comparable to the use of a logger, vaginal temperature should be assessed twice a day with a thermometer [7]. When it is not possible to have a temperature value every 12 h, but temperature can be measured only once a day, it should be evaluated in the time interval from 20:00 to 1:00. In this case, indications about parturition timing are comparable to the use of a vaginal logger with a sensitivity range of 69.2–84.6% and a specificity range of 88–95.8%. A temperature drop greater than or equal to 0.4 °C within 24 h indicates that parturition will start within 48 h with 69% sensitivity and 88% specificity [7]. This means that in 3 out of 10 cases, it is not possible to correctly detect the temperature drop, but when this is evident, it is a reliable indicator of parturition in the following two days. Therefore, it should not be used alone for the prediction of parturition date or planning a C-section.

### 3.5. Other Maternal Markers Studied for Predicting the Day of Parturition

Canine reproductive endocrinology around parturition has been studied because of the peculiarities of the canine species [30,31], and recently some hormonal parameters other than P4 have been studied because of their valuable potential for delivery prediction. Serum cortisol concentration increases in late pregnancy around parturition, and it reaches a plateau 12 h after the onset of parturition, declining to baseline values up to 36 h after parturition [5,31]. However, a correlation between cortisol and PGF2alpha, or P4 concentration during the last 48 h prior to parturition was not established [5]. Moreover, it seems not to be a reliable parameter for the prediction of cervical dilatation timing [36]. A recent study, aimed at assessing endocrine peripartum changes by measuring concentrations of a wide panel of steroids, found significant variations for cortisone concentrations on the day before parturition [43], but the large variability of concentration between bitches together with the timing of its increase may point out a peripartal maternal stress more than the onset of labor. More studies on the topic could bring more insight to discriminate corticosteroids increase due to the approaching of parturition instead of maternal stress. Serum acute phase protein concentrations have been studied extensively as diagnostic markers during early pregnancy. The correlation between serum C-reactive protein and labor onset has been considered [44], but a late significant variation of just 24 h prepartum, together with a low specificity, make it unsuitable for the prediction of the day of parturition.

## 4. Embryonic and Fetal Parameters

### 4.1. Development of Embryonic and Extra-Embryonic, Fetal and Extra-Fetal Structures, and First Ultrasonographic Detection of Organs

The study of canine embryonic and fetal development has greatly benefitted from the use of US, as early detection of anatomical structures is fundamental in staging pregnancy, and therefore predicting the parturition day. Many embryonic and fetal structures can be viewed on US during their development, from embryonic vesicles in early pregnancy to the fetal intestine during the last week of pregnancy. The day/s at which each structure or organ become visible on US for the first time is relevant from a clinical point of view—as it is correlated with gestational age and indirectly to the day of parturition—and is defined as first ultrasonographic detection (FUD) [11,45,46,47], usually reported in relation to the preovulatory LH peak [48] or to the estimated day of ovulation [49].

Identifying FUD of embryonic and fetal structures/organs is often not feasible in a clinical setting, as it is a time-consuming procedure that requires sequential US monitoring. For most embryo-fetal structures, there is a general consensus on time of FUD, although this is frequently a range rather than a single day [15,46,48,49].

Table 1 and Table 2 depict all the most described embryonic and fetal structures reported in the literature, with the first appearance interval and the authors’ reference.

### 4.2. Measurement of Embryonic, Extra-Embryonic, Fetal and Extra-Fetal Structures

When mating or ovulation data are not available, the ultrasonographic measurement of fetal and extra-fetal parameters is fundamental in order to stage pregnancy and estimate the parturition day. The most studied extra-embryonic and extra-fetal parameters include inner chorionic cavity (ICC), outer uterine diameter (OUD) and placental thickness. Embryonic and fetal parameters include crown-rump length (CRL), body diameter (BD), biparietal diameter (BP), deep portion of diencephalo-telencephalic vesicle (DPTV) and kidney length. Data obtained by measuring these structures have been used to calculate formulas and tables for the determination of gestational age or the number of days before parturition (dbp), which can be used in practice to stage pregnancy when ovulation is unknown.

Formulas for the determination of gestational age or of dbp are normally based on a linear or polynomial regression analysis of repeated measures throughout pregnancy and are generally regarded as useful in clinical practice. Tables combining regression of fetal trunk and fetal BP, as well as tables for ICC to determine parturition timing, have been reported [46,55], although they are less commonly used in clinical practice [11].

Ultrasonographic measurements should be done on at least two fetuses located in both uterine horns [16,17,18,51]. In singleton pregnancies, multiple parameters should be considered for a more accurate parturition timing [12].

In small- (≤10 kg) and giant- (>40 kg) size bitches, maternal body weight influences the accuracy of ICC, CRL and BD for the determination of parturition time [6]. This bias may be corrected using formulas elaborated specifically per classes of weight. When using tables calculated by Yeager et al. [48] and England et al. [46], the above bias may be minimized by adding one day to and subtracting two days from the predicted parturition day for small and giant size bitches, respectively [6]. Independently of bitch size, litter size does not affect ICC, CRL and BD [6].

#### 4.2.1. Inner Chorionic Cavity, Outer Uterine Diameter, Placental Thickness

Inner chorionic cavity and outer uterine diameter are normally measured in early pregnancy, while placental thickness may be used also in mid to late pregnancy. The values of these parameters are used with body maternal size specific formulas to calculate gestational age or dbp.

Outer uterine diameter is measured from the outer points of the implantation site considering two orthogonal diameters of the gestational vesicle, which should be scanned when it appears as a round shape and reaching its larger diameter [38]. OUD leads to inaccurate parturition timing, because of its not well defined measurement markers [56]. The measurement of ICC is done using the same scanning planes but measuring the distance between the inner sides of the chorionic wall from trophoblastic decidual reaction sites [14,57]. ICC is the most accurate extra-embryonic parameter in early pregnancies [11,12], and it is useful from days 17–18 to 37 after ovulation, with its best accuracy being on days 20–25 [6,11,51,55]. In small- and medium-size bitches ICC is reported to have an accuracy of 64–91% within ±1 day, and in large-size bitches an accuracy of 85–88% within ±2 days in the prediction of parturition day [14,55,56].

The reliability of the placental ultrasonographic measurements for prediction of parturition day is controversial [12,22,55,56,58]. Zonary placental length, measured as the distance between the two extremities of the zonary placenta in a longitudinal scan, is not significantly correlated with gestational age in small-sized dogs [55], while placental thickness is positively correlated with gestational age, regardless of breed and body weight [58]. Formulas for ICC, OUD and placental thickness, along with the time of pregnancy at which they were calculated, are reported in Table 3.

#### 4.2.2. Crown-Rump Length, Body Diameter, Biparietal Diameter, Deep Portion of Diencephalo-Telencephalic Vescicle

Crown-rump length can be measured only from 26 to 45 days after LH peak, as afterwards, the fetal body starts to change its shape by flexing, making CRL measurement less accurate [22,46]. The highest accuracy of CRL measurement for the determination of gestational age is reported on day 30 of pregnancy [6,22]. It is measured as the distance between the most rostral point of the fetal head and the most caudal point of the perineal area using a straight line, when the fetus appears in its entire length [22]. Some authors consider the use of CRL for predicting parturition time inaccurate, because of the lack of a linear and significant correlation with gestational age [55].

In the second half of pregnancy, BD is measured by drawing two orthogonal lines at the level of the largest section of the fetus using a transversal scanning plane, in which both the liver and stomach must be included [22]. BD may be measured from day 26 after LH peak, with its best accuracy being on day 30 of pregnancy [6].

In late pregnancy, BP is the most accurate fetal parameter for predicting parturition time [22]. BP may be identified as earlier as day 30 of pregnancy, although a better visualization of parietal bones may occur after day 35 [6]. BP is measured by drawing a line in the same longitudinal scan as that for CRL and in a frame in which the falx cerebri is visualized and the two parietal bones are parallel [22]. BP has a good accuracy during the 5th and 6th week of pregnancy (95.2% and 88.4%, within ±2 day, respectively), although it is regarded as a reliable parameter until the 8th week (85.3%, ±2 days) [17]. BP should not be used during the last week of pregnancy for parturition timing to plan a C-section because of its low accuracy (50.9% within ±1 day and 69.8% within ±2 days) and high variability of measurements among fetuses in the same and different litters [17,19].

The combined use of ICC in early pregnancy and BP in late pregnancy has been studied, showing no statistically significant differences in the prediction of parturition day (within ±1 and ±2 days), except for small-size bitches (78.9% and 42.3% within ±1, respectively) [14,61]. When litter size is larger or smaller than the normal reference for maternal size, ICC is reported to be more accurate than BP (83.3% vs. 68.4% for larger litter size, 88.9% vs. 84% for smaller litter size, ICC vs. BP, respectively) [14,15]. In any case, both ICC and BP could be measured from 35 to 25 dbp [56], and they have similar accuracies during the 5th week of pregnancy [17].

Diencephalo-telencephalic vesicle may be visualized in late pregnancy from 30 to 7 dbp, with the same scan used for BP [16,22]. The best day for DPTV measurement is 7 dbp, because of the increased consistency of its measurement and the lower standard deviation on this date [16]. DPTV is significantly and linearly correlated with gestational age. The accuracy of DPTV for the prediction of parturition day is of 42.9% within ±1 day and of 62% within ±2 days, with no differences between sizes. Furthermore, its accuracy is higher in normal (two to six puppies in small- and five to nine puppies in medium- and large-size bitches) and large litter sizes than in small ones (62.3%, 73.7% and 47.6% within ±2 days, respectively), and it seems to be unaffected by fetal sex ratio [16]. In Table 4, an overview of formulas of BP, BD, CRL, DPTV and the time at which they were calculated is reported.

#### 4.2.3. Kidney Length

Recently, fetal kidney length has been studied, and it has been reported to be strongly and positively correlated with gestational age [13]. The proposed formula (Table 4) may be applied from 24 to 1 dbp, and the best period for monitoring kidney length is from 15 to 11 dbp, at which time accuracy, sensitivity and specificity are 99.98%, 99.95% and 99.92% within ±1 day, respectively. More studies on the influence of breed and body weight, about specific formulas for different body sizes and about the differences in length of the right and left fetal kidney are needed [13].

#### 4.2.4. Fetometric Formulas for Specific Breeds or Maternal Sizes

Two different approaches have been reported for giant size breeds. Socha et al. [59] proposed the use of ICC and BP formulas for medium-size bitches without any correction factor, with an overall accuracy of 76.66% (±1 day) and 90% (±2 days) for ICC and of 54.16% (±1 day) and 79.16% (±2 days) for BP. Alonge et al. [18] formulated specific equations for large- and giant-size bitches, with an overall higher accuracy than those for medium-size bitches (83.1% ± 2 days for ICC and 88.3% ± 2 days for BP for large breeds and of 100% ± 2 days for ICC and 87% ± 2 days for BP for giant breeds, respectively). These formulas are not influenced by fetal sex ratio. In giant-size bitches, the BP accuracy is lower than ICC (84.6% and 100% within ±2 days, respectively), and it is affected by litter size, with the highest accuracy (96.4% within ±2 days) found between five and nine puppies [18].

Specific formulas for ICC and BP have also been calculated for miniature-size dogs (≤5 kg) [60]. In contrast with what reported for breeds of other maternal size, BP seems to be more accurate than ICC in the prediction of parturition day [60].

Because of differences in head and body morphology and gestation length between different breeds included in the same size class, specific breed formulas have been proposed in German Shepherd [62,63], Golden Retriever [64], Chihuahua [57] and Yorkshire breeds for the most commonly used parameters (ICC, BP, BD, CRL and DPTV) to increase the accuracy of parturition timing (Table 5) [64]. Prediction tables have also been calculated for Maltese and Yorkshire breeds [55].

Breed-specific formulas in German Shepherd bitches showed an accuracy of 94.5% ± 2 days for ICC and 91.7% ± 2 days for BP, respectively [62]. The highest accuracy was obtained when ICC and BP were measured between 23 and 25 and between 45 and 48 days after ovulation, respectively [63]. BD was found to be more accurate than CRL and DPTV [63]. Breed-specific formulas for Yorkshire Terriers and Golden Retrievers were [64] compared to the small- and medium-size dog formulas obtained by Luvoni and Grioni [56]. The authors found no statistical differences between formulas, with the exception of Golden Retrievers that had a more accurate prediction obtained with BP specific formula, compared to that of medium-size bitches [64].

Socha and Janowski [65] compared the use of medium-size bitch formulas [56] with formulas specific for German shepherd dogs [62]. They reported that the ICC formula for medium-size bitches had a better accuracy than the breed-specific formulas, and that the BP formula for German Shepherds [62] was more accurate than the BP formula for medium-size bitches. This could be due to the fact that ICC is less influenced by breed, while BP depends on the shape of head that is characteristic for each breed.

## 5. Evaluation of the Fetal Maturity

### 5.1. Evaluation of the Fetal Gastro-Intestinal Tract Motility and of Kidney Development

Fetal gastrointestinal development was recently studied by Gil et al. [54]. The intestinal area may be visualized caudally to the fetal liver as a homogeneously echogenic region from 40 to 44 days of pregnancy (23–19 dbp), the intestinal wall may be visualized in some intestinal portions from 44 to 48 days (19–15 dbp). The intestinal wall and its mucosal surface may be easily identified from day 50 to day 54 (13–9 dbp), when gastrointestinal motility appears for the first time. In this stage of pregnancy, the US identification of peristalsis requires a prolonged (at least one minute) observation and may only be seen in some intestinal portions. All the intestinal wall layers become visible from day 59 to 62 of pregnancy. At this time a distinction between the surface of the mucosa and the intestinal wall may be detected, and a clear gastrointestinal peristalsis is observed after 3 s of observation. In one study, gastrointestinal motility was rapidly detected along the whole gastrointestinal tract between 4–1 dbp, associated with the presence of gastrointestinal mucous and fluid content [54]. The usefulness of fetal gastrointestinal motility for fetal maturity was studied during the last 10 days of pregnancy [41], at which time an increase of fetuses showing gastrointestinal motility was observed, from 17.1% to 63.3%, in the last five days prepartum. Fetal gastrointestinal motility showed a weak negative correlation with vaginal temperature during the last 10 days of pregnancy [41].

Fetal kidney development during the last trimester has been studied and well described in dogs. US kidney changes observed during the last 5 days of pregnancy are not indicative of imminent parturition, while the observation of fetal gastrointestinal motility confirms that renal development is complete [13].

### 5.2. Fetal Lung Maturation

Fetal lung maturity is an essential requirement for extra-uterine fetal survival. Canine fetal lung development has been studied from both anatomic and ultrasonographic points of view [53,66]. Type I and type II pneumocytes develop in the canalicular phase between 48 and 57 days of pregnancy [66]. The saccular phase is the phase in which the production of surfactant produced by type II pneumocytes begins in humans; for this reason, it has been postulated that, in the canine species, it probably also occurs in the saccular phase, from day 57 to 60. From day 57 onwards, canine fetal lungs could theoretically be ready for extra-uterine life. The alveolar phase, which is the last lung developmental phase, occurs during the neonatal period [66]. Fetal lung echogenicity has been analyzed quantitatively through the assessment of mean grey level, which rapidly increases from days 49 to 56, reaching a plateau at 57–63 days post ovulation [53]. Therefore, it has been proposed that lung-to-liver ratio of mean grey level could be used as an accurate parameter to evaluate fetal lung maturity, with 83% specificity and sensitivity [53], due to its significant decrease during the last week before parturition. This finding, together with a lack of studies aimed at analyzing the echogenicity of different fetal structures, underlines the need for further research to assess the value of quantitative analysis for fetal maturity evaluation, because at the moment it is not possible to plan a C-section based on lung-to-liver ratio mean grey level.

## 6. Ultrasonographic Assessment of Maternal and Fetal Heart Rate and Blood Flow

### 6.1. Fetal Heart Rate (FHR), and Feto-Maternal Heart Rate (FHR/MHR) Ratio

Fetal heart rate (FHR) is routinely assessed and monitored throughout canine pregnancy using pulsed-wave Doppler [8,11,12,67]. Normal FHR is >220 beats per minute (bpm); values of 180–220 bpm may indicate moderate fetal distress, whereas values < 180 bpm indicates severe fetal distress [8,11,51,67]. When FHR is <160 bpm, intervention is needed. Fetuses with a FHR < 130 bpm should be delivered within 1–2 h and high mortality rate is expected when fetuses with < 100 bpm are not promptly delivered [3,67]. An increase in FHR and feto-maternal heart rate (FHR/MHR) ratio from 35 dbp is described, reaching its maximum value on 20 dbp, and followed by a progressive decrease prepartum [68]. This pattern is caused by the development of fetal sympathetic nervous system control prior to the development of the parasympathetic nervous system [12,68]. During prepartum, transient FHR decelerations followed by accelerations have been described from 5 dbp onwards [8,69]. These variations are likely due to uterine contractions, as reported for human fetuses by means of cardiotocography [8]. Gil et al. [8] detected physiological FHR variations 72 h prior to parturition in some fetuses, and in all fetuses within 6 to 1 h prior to parturition. In a recent study, a specific parameter for FHR variations, which is the heart rate (HR) variation parameter, was calculated (HR variation = FHR gradient (maximum FHR − minimum FHR) × 100/maximum FHR) [69]. A HR variation value >30% is predictive of parturition within 12 h with a sensitivity of 88% and a specificity of 50% [69].

While evidence has accumulated on the role of fetal hypoxia, impending parturition, gestational age and fetal health status on FHR, much less is known on other factors which may influence FHR, such as maternal pregestational bodyweight, fetal movements and litter size [12,68]. The roles of these last three factors need to be further investigated. FHR recorded in a group of bitches from 5.8 to 68 kg was reported to be higher in bitches with high and low bodyweight compared to medium-bodyweight bitches [68]. A similar difference in the FHR values depending on different maternal size was not confirmed in a recent study by Blanco et al. [70]. Prolonged or repeated measurements of FHR in the same US session and a comparison between fetuses of the same litter should be performed in order to properly assess transient variations of FHR.

### 6.2. Uterine Artery Flow

Uterine artery perfusion increases in the last two trimesters of pregnancy, and the indices associated with blood flow velocities (resistive index, RI and pulsatility Index, PI) decrease as gestational age advances [12,71,72]. Peak systolic (PSV) and end diastolic velocities (EDV), but not RI, are affected by litter size [73]. An equation describing the correlation between uterine artery RI and gestational age has been calculated for small-size bitches, together with a RI reference range values during the second half of pregnancy [73]. Variations in the previously described parameters are due to increased fetal needs for blood flow as pregnancy advances. During mid-late pregnancy, an influence of litter and maternal size on RI is reported, with lower values observed in large-size bitches compared with small-size bitches [70]. Another change evident in blood flow is the early diastolic notch of the uterine artery which is reported to disappear at 16 ± 5 dbp. Serial US examinations are always preferable. Uterine artery flow studies do not need to be performed always on both sides as right and left uterine arteries produce similar values for RI and PI in any day of pregnancy [72]. Nowadays, variations in the uterine artery flow could give just an indication in staging pregnancy. Further studies are needed to use indices related to uterine artery flow for the prediction of parturition date.

### 6.3. Umbilical Artery Flow

The umbilical artery has different waveforms throughout pregnancy: a systolic component is reported from week 4 to 6 of gestation, while a diastolic component may also be observed later on [71,74]. The early diastolic notch of the umbilical artery disappears at 21 ± 1 dbp [72]. Umbilical artery RI could be useful for parturition timing and early fetal distress assessment [10]. Giannico et al. [10] reported that normal parturition is likely to occur within 12 h when all fetuses show a RI < 0.7. An influence of litter size and maternal size on umbilical RI is reported in mid-late pregnancy [70]. HR variation and umbilical artery RI are correlated, and their simultaneous monitoring could be useful for predicting the day of parturition. When a HR variation value >30% and a RI < 0.7 are recorded, parturition is expected within 12 h, with a sensitivity of 95% and a specificity of 80% [11,69].

## 7. Discussion and Conclusions

In this review, we illustrated the parameters commonly used in canine practice and described in the scientific literature to assess fetal readiness for parturition. This concept links together two different actors: dam and conceptus. The maternal side, where hormonal changes play an important role in driving prepartum events as well as parturition, can be effectively monitored through changes in serum P4 concentration and vaginal cytology. The more reliable way to manage a canine pregnancy is to assess ovulation time or to detect the D1, and monitor pregnancy period using clinical parameters, US as well as serum P4 assay until its concentration drops to values lower than 2.0 ng/mL as an indication of the approaching onset of parturition [11]. Surprisingly, despite its frequent clinical use, not all authors agree in the prognostic value of rectal temperature for parturition timing in dogs. Further studies on sensitivity and specificity of vaginal and rectal temperatures are needed. Fetal development has mostly been studied through US imaging of fetal structures, and either described through the first identification of specific structures, or through a formula that correlates specific structure size with gestational age or with the day before parturition [11,12,48]. Fetometry is considered a useful tool; however, not all fetometric parameters accurately indicate the parturition day throughout different stage of pregnancy, and particularly, no clear correlation has been shown between any parameter and parturition day during the last week of pregnancy [17]. For this reason, there is still demand for determining parameters in the last seven days of pregnancy that can be useful and accurate for predicting parturition. Fetal maturity evaluation has recently been proposed as a possible new field of study, which relies on quantitatively measurement of lung echogenicity and/or gastrointestinal motility as proof of complete fetal organ maturation [41,53,54]. Recent data on these parameters show that their combined evaluation may allow accurate assessments of fetal readiness for parturition during the last five days of pregnancy. Further studies related to factors influencing these parameters, such as correlations with hormonal changes, maternal size, or breed, are still needed in order to choose the best day for a C-section.

However, a question arises: what are the most reliable parameters that can be used when the day of ovulation is unknown? It is difficult to extrapolate data from the literature to answer this question, because sometimes comparing different studies can be difficult for the following reasons:

(1) differences in the method of serum P4 assay used;
(2) differences in comparing values of gestational age with days before parturition; although these values should be highly correlated, to our knowledge, no study on this correlation has been done yet between counting gestational age from the day of ovulation or the day of LH peak vs. days before parturition;
(3) many, but not all, of the factors known to influence pregnancy length, such as breed, maternal size and litter size, have been taken into account when using US measurements. Some authors have tried to overcome these difficulties by building breed-specific formulas, a recommendable approach which, however, requires more insight regarding comparisons among breeds, as well as clinical testing. Specific breed formulas should be used whenever available; however, as no accuracy has been reported for almost any of them, more clinical data on specific breed formulas are needed before they can be recommended in clinical practice;
(4) nearly all studies evaluate only one or two parameters at a time, whereas more parameters are commonly evaluated in clinical practice. The most appropriate combination of parameters used for determining gestational age and organ development detected by US has not been established yet. As an example, the use of FHR/MHR ratio may be more accurate and helpful than FHR alone for fetal health assessment [68], and even though an equation has been calculated, evidence for its usefulness in clinical settings and correlation with other parameters still has to be provided.

Currently, the only two studied and reliable combinations of parameters are (a) the sequential measurement of ICC in early (42–21 days before parturition) and BP in late (37–1 days before parturition) pregnancy [14,61]; and (b) HR variation and RI during the last 5 days of pregnancy [69]. Thus, further studies with adequate case numbers and considering maternal and fetal parameters as well as the effect of maternal and litter size are needed to understand the interactions among them.

Moreover, further studies are necessary in order to better describe the use of US measurements to assess fetal maturity. Indeed, fetal gastrointestinal motility seems to be a useful parameter for assessing fetal maturity during the last week of pregnancy [41], but assessing its predictive value requires further studies with larger sets of samples. The use of any US parameter without a parallel P4 assay is strongly discouraged when ovulation day is unknown, as the current evidence suggests that US alone is not a safe way to establish the day of parturition. It would also be useful to investigate the use of US to evaluate fetal maturity when a C-section is planned using aglepristone; in this case, US evaluation may help to correlate the hormonal variations induced by aglepristone with the changes of US fetal maturity parameters and the outcomes of planned C-sections.

In conclusion, many hormonal and ultrasonographic parameters indicating maternal and fetal readiness for parturition are currently available, the majority of which show good accuracy. More research is needed to develop a panel of US parameters throughout the entire pregnancy that are accurate enough to foresee the day of onset of parturition. Thus, setting up specific combinations along with their timing of use during pregnancy may be of help in the accurate prediction of parturition day, and this may help all practitioners facing obstetrical cases in making accurate forecasts regarding the results of their protocols and the time of delivery.

## Figures and Tables

**Table 1:** Days of first appearance at ultrasound of embryonic and extra-embryonic structures observed during the first half of pregnancy and references.

Embryonic and Extra-Embryonic Structures	First Detection (Days After Ovulation)	References
Gestational sac	17–19	[49]
	18	[11,48]
Embryo	23	[15,50]
	22–23	[11,49]
Heartbeats	23–24	[49]
	21–23	[47,50,51]
	23	[48]
Zonary placenta	23–26	[49]
	25–28	[48]
Embryo bipolarization	27–30	[49]
	23–26	[47]
Anechoic area in head	29–33	[49]
	25–29	[48]
Limb buds	27–31	[49]
	32–33	[50]
	31–33	[47,48]
Movements	32–34	[47,48,49]

**Table 2:** Days of first appearance at ultrasound of fetal structures observed during the second half of pregnancy reported in literature and references.

Fetal Structures	First Detection (Days After Ovulation)	References
Stomach	29–33	[11,49]
	34–37	[48]
Urinary bladder	31–35	[49]
	33–37	[48]
Skeletal structures ^1^	29–33	[11]
	34–38	[49]
	31–37	[48]
	33	[50]
DPTV ^2^	29–33	[52]
Abdomen/thorax distinction	34–36	[11,53]
Fluid filled stomach	34–36 (90% of fetuses)	[46,51]
Lung hyperechoic vs. liver	35–38	[11]
	34–36	[49]
	36–40	[15,48]
Liver hypoechoic vs. abdomen	35–38	[49]
	37–45	[48]
Kidney	41–43	[49]
	37–45	[48]
	20–24 dbp ^3^	[13]
Corticomedullary definition	20–16 dbp ^3^	[13]
Eyes	37–45	[48]
Gastrointestinal tract	15–19 dbp ^3^	[54]
Gastrointestinal luminal content	9–13 dbp ^3^	[54]

^1^ skeletal structures: visible as hyperechoic structures, head and spinal column could be firstly visualized; ^2^ DPTV = deep portion of diencephalo-telencephalic vesicle; ^3^ dbp = days before parturition.

**Table 3:** Formulas for calculations of days before parturition (dbp) or gestational age (ga: days after LH peak) in bitches of different size (miniature ≤ 5 kg, small ≤ 10 kg, medium 11 to 25 kg, large 26 to 40 kg, and giant > 40 kg [16,18,59]) for inner chorionic cavity (ICC), outer uterine diameter (OUD) and placental thickness. The measurements (scale either in mm or cm) are the mean of ICC and OUD orthogonal measurements in all measured fetuses. References are reported in brackets. Time of measurement refers to the dbp or days of pregnancy at which each parameter was calculated. When available, dbp or week of pregnancy in which accuracy was ≥80% (±1 or ±2 days) is also reported.

Parameter	Maternal Size	Formula	References	Time	Accuracy ≥80% at
ICC	miniature	dbp = (0.62887 × mm) − 44.04	[60]	41–26 dbp	no data
	small	dbp = (mm − 68.68)/1.53	[22,56]	42–21 dbp	42–21 dbp (±1 day) [56] 4–5 week of pregnancy (±2 days) [17]
	medium	dbp = (mm − 82.13)/1.8	[22,56]	42–21 dbp	42–21 dbp (±1 day) [56] 4–5 week of pregnancy (±2 days) [17]
	large	dbp = (mm − 105.1)/2.5	[18]	42–26 dbp	42–26 dbp (±2 days) [18]
	giant	dbp = (mm − 88.1)/1.9	[18]	40–25 dbp	40–25 dbp (±2 days) [18]
OUD	small	dbp = (mm − 85.17)/1.83	[22,56]	42–27 dbp	no data
	medium	dbp = (mm − 80.78)/1.57	[22,56]	42–27 dbp	no data
Placental thickness	small	dbp = (mm − 5.8)/0.12	[22,56]	42–21 dbp	no data
	medium	dbp = (mm − 18.99)/0.45	[22,56]	42–21 dbp	no data
Placental thickness	all size bitches	ga = (cm + 0.314)/0.021	[58]	20–60 days of pregnancy	no data

**Table 4:** Formulas for calculations of days before parturition (dbp) or gestational age (ga: days after LH peak) in bitches of different size (miniature ≤ 5 kg, small ≤ 10 kg, medium 11 to 25 kg, large 26 to 40 kg, and giant > 40 kg [16,18,59]) for crown-rump length (CRL), body diameter (BD), biparietal diameter (BP), deep portion of diencephalo-telencephalic vesicle (DPTV) and kidney length. The measurements (scale either in mm or cm) are the mean of BD orthogonal measurements in all measured fetuses. References are reported in brackets. Time of measurement refers to the dbp or days after LH peak in which each parameter was calculated. When available, dbp or week of pregnancy in which accuracy was ≥80% (±1 or ±2 days) is also reported.

Parameter	Maternal Size	Formula	References	Time	Accuracy ≥80% at
CRL	Beagles	ga = 24.64 + 4.54 × cm − 0.24 × cm^2^	[22,48]	23–48 days after LH peak	no data
BD	Beagles	ga = 22.89 + 12.75 × cm − 1.17 × cm^2^	[22,48]	23–60 days after LH peak	no data
BP	miniature	dbp = (1.6190 × mm) − 39.70	[60]	23–6 dbp	no data
	small	dbp = (mm − 25.11)/0.61	[22,56]	37–1 dbp	5–8 week of pregnancy (±2 days) [17]
	medium	dbp = (mm − 29.18)/0.7	[22,56]	37–1 dbp	5–8 week of pregnancy (±2 days) [17]
	large	dbp = (mm − 30)/0.8	[18]	30–2 dbp	30–2 dbp (±2 days) [18]
	giant	dbp = (mm − 29)/0.7	[18]	35–1 dbp	35–1 dbp (±2 days) [18]
BD and BP	Golden R. ^1^ Labrador R. ^1^	dbp = 34.27 – 5.89 × BPcm − 2.77 × BDcm	[22,46]	from 20 dbp-to parturition	39–62 days after LH peak < 80% (±2 days) [6]
DPTV	small	dbp = (mm − 10.11)/0.24	[16,52]	30–8 dbp	30–8 dbp (±2 day) [52]
	medium	dbp = (mm − 14.15)/0.4	[16,52]	30–8 dbp	<80% 30–8 dbp (±2 days) [52]
	large	dbp = (mm − 10.27)/0.24	[16]	25–2 dbp	<80% 25–2 dbp (±2 days) [16]
Kidney length	3–26 kg	dbp = 27.414 − 11.771 × cm	[13]	24–1 dbp	15–11 dbp (±1 day) [13]

^1^ R. = Retriever.

**Table 5:** Specific formulas for calculations of days before parturition (dbp) in different breeds (German Shepherd, Yorkshire Terrier, Golden Retriever and Chihuahua) for inner chorionic cavity (ICC), crown-rump length (CRL), body diameter (BD), biparietal diameter (BP), deep portion of diencephalo-telencephalic vesicle (DPTV). Using these formulas, dbp could be calculated. The measurements (scale either in mm or cm) are the mean of ICC and BD orthogonal measurements in all measured fetuses, respectively. References are reported in brackets. Time of measurement refers to the dbp or days after LH peak in which each parameter was calculated. When available, dbp in which accuracy was ≥80% (±1 or ±2 days) is also reported.

Parameter	Formula	References	Time	Accuracy ≥80% at
Specific formulas for German shepherd dog				
ICC	dbp = 44.76 − (4.34 × cm)	[62]	day 23–37 after LH peak	42–27 dbp (±2 days) [65]
BP	dbp = 38.65 − (12.86 × cm)	[62]	day 43 after LH peak-to parturition	28–4 dbp (±1 days) [65]
BD	dbp = −34.92 + (5.41 × cm)	[63]	33–2 dbp	33–2 dbp (±2 day) [63]
CRL	dbp = −35.41 + (2.10 × cm)	[63]	38–19 dbp	38–19 dbp (±2 day) [63]
DPTV	dbp = −33.56 + (28.38 × cm)	[63]	27–9 dbp	27–9 dbp (±2 day) [63]
Specific formulas for Yorkshire Terrier				
ICC	dbp = (mm − 74.68)/1.75	[64]	40–25 dbp	no data
BP	dbp = (mm − 24.5)/0.62	[64]	25–0 dbp	no data
Specific formulas for Golden Retriever				
ICC	dbp = (mm − 84.66)/1.86	[64]	40–25 dbp	no data
BP	dbp = (mm − 31.19)/0.8	[64]	25–0 dbp	no data
Specific formula for Chihuahua				
BP	dbp = −15.46 × cm + 38.72	[57]	25–0 dbp	no data

## Data Availability

Data sharing not applicable.
